# 固相萃取-超高效液相色谱-串联质谱法测定发酵食品中的曲酸

**DOI:** 10.3724/SP.J.1123.2022.10002

**Published:** 2023-07-08

**Authors:** Dongyang CHEN, Hao ZHANG, Lei ZHANG, Yihong WANG, Xiaodan WANG, Jiali FENG, Jing LIANG, Xuan ZHONG

**Affiliations:** 1.湖南省疾病预防控制中心,湖南长沙410005; 1. Hunan Provincial Center for Disease Control and Prevention, Changsha 410005, China; 2.国家食品安全风险评估中心,北京100022; 2. China National Center for Food Safety Risk Assessment, Beijing 100022, China

**Keywords:** 固相萃取, 超高效液相色谱-串联质谱法, 曲酸, 发酵食品, solid-phase extraction (SPE), ultra performance liquid chromatography-tandem mass spectrometry (UPLC-MS/MS), kojic acid, fermented foods

## Abstract

曲酸由曲霉属和青霉属真菌好氧发酵产生,天然存在于发酵食品中,且常作为防腐添加剂滥用于食品工业中,监测其在发酵食品中的含量及评估其暴露水平带来的健康风险具有重要意义。本研究针对发酵食品基质复杂、基质干扰严重这一关键瓶颈,通过溶剂转换、固相萃取净化,有效地去除了基质干扰。通过系统优化固相萃取条件和仪器条件,建立了发酵食品中曲酸含量的固相萃取-超高效液相色谱-串联质谱(SPE-UPLC-MS/MS)测定方法。样品经0.1%甲酸无水乙醇溶液超声提取,氮吹至近干后用超纯水复溶,过PRiME HLB固相萃取小柱,在ACQUITY UPLC^®^ BEH C_18_色谱柱(100 mm×2.1 mm, 1.7 μm)上分离,以甲酸-5 mmoL/L乙酸铵(1∶999, v/v)溶液和甲酸-乙腈(1∶999, v/v)溶液作为流动相进行梯度洗脱,电喷雾正离子模式下以多反应监测扫描方式测定,内标法定量。在优化的测定条件下,曲酸在5 min内可获得有效分离,在5.0~100.0 μg/L内线性关系良好,相关系数(*r*)为0.9994,方法检出限为2~5 μg/kg,定量限为6~15 μg/kg。在低、中、高3个加标水平下的回收率为86.8%~111.7%,日内精密度为1.0%~7.9%(*n*=6),日间精密度为2.7%~10.2%(*n*=5)。通过建立的方法对酱油、醋、酒、酱、豆豉、腐乳等重点食品中的曲酸含量进行测定,发现醋的检出率最高,其次为酒、酱、酱油、豆豉和腐乳,曲酸含量为5.69~2272 μg/kg。该方法具有高效、准确、灵敏等特点,可为发酵食品中曲酸含量的日常监测提供可靠的技术支持。

## Abstract (en)

Kojic acid naturally appears in fermented foods and can be formed during the aerobic fermentation process induced by *Aspergillus* and *Penicillium fungi*. It is widely used in the food industry because it exhibits antibacterial and antifungal properties and does not affect food taste. However, recent studies indicate that kojic acid may be a potential carcinogen. Therefore, assessing the health risks of kojic acid in fermented foods are of great importance, and developing a sensitive and accurate analytical method for this compound is a significant endeavor. Much efforts have been devoted to the detection of kojic acid using electrochemistry, high performance liquid chromatography (HPLC), gas chromatography-mass spectrometry (GC-MS), and high performance liquid chromatography-tandem mass spectrometry (HPLC-MS/MS). HPLC and HPLC-MS/MS are the analytical techniques most often employed for this purpose. Of these two methods, HPLC-MS/MS displays excellent sensitivity and is the optimal selective technique. Pretreatment is usually necessary for kojic acid determination because of the complex matrix effects of fermented foods. However, few researches on the determination of kojic acid in food are available, and, to the best of our knowledge, the determination of kojic acid using solid-phase extraction (SPE) pretreatment has not been reported yet. Herein, a convenient, sensitive, and accurate method was developed to determine kojic acid in fermented foods using solid-phase extraction-ultra performance liquid chromatography-tandem mass spectrometry (SPE-UPLC-MS/MS). The pretreatment conditions, such as the extraction solvent, cartridge, rinse solvent, and eluent, were systematically optimized. The samples, including soy sauce, vinegar, liquor, sauce, fermented soya bean, and fermented bean curd, were extracted with 0.1% formic acid-absolute ethyl alcohol and purified using a PRiME HLB cartridge. Kojic acid was separated using an ACQUITY UPLC^®^ BEH C_18_ column (100 mm×2.1 mm, 1.7 μm) with formic acid-acetonitrile (1∶999, v/v) and formic acid-5 mmol/L ammonium acetate (1∶999, v/v) solutions as the mobile phases under gradient elution mode. MS was performed in electrospray positive ionization (ESI^+^) and multiple reaction monitoring (MRM) modes. An internal standard method was used for quantification. Under optimized conditions, good linearity was achieved at mass concentrations of 5.0-100.0 μg/L, with a correlation coefficient (*r*) of 0.9994. The limits of detection and quantification of the method for kojic acid were 2-5 μg/kg and 6-15 μg/kg, respectively. Good recoveries of 86.8%-111.7%, intra-day precisions of 1.0%-7.9% (*n*=6), and inter-day precisions of 2.7%-10.2% (*n*=5) were also obtained. The matrix effect was evaluated by establishing a matrix-matching calibration curve, and weak inhibitory effects were found in vinegar and liquor; moderate inhibitory effects in fermented bean curd, fermented soya bean, and soy sauce; and a strong inhibitory effect in sauce. The developed method was used to detect kojic acid in 240 fermented foods, and the results showed that the detection rate of vinegar was the highest, followed by liquor, sauce, soy sauce, fermented soya bean, and fermented bean curd, the contents were 5.69-2272 μg/kg. Matrix interferences can be significantly reduced by optimizing the pretreatment and detection procedures. The proposed method is sensitive, accurate, and can be used to analyze kojic acid in fermented foods.

曲酸是曲霉属和青霉属真菌好氧发酵产生的有机酸,又名曲菌酸,为杂环类化合物,具有抑菌和抗氧化功能^[1]^,其在食品中可起到防腐、保鲜和护色等作用^[2]^。曲霉属真菌在传统发酵食品中使用广泛,许多以曲霉发酵的产品天然存在曲酸^[3]^;且曲酸作为添加剂不影响食品的口感和质感^[4]^,因此常作为防腐添加剂用于食品工业中。近年来的科学研究表明曲酸可能具有潜在的致癌性^[5,6]^,其安全性成为行业关注的焦点。我国并未将曲酸列入《食品安全国家标准 食品添加剂使用标准》(GB 2760-2014)中,日本禁止曲酸作为食品添加剂使用,但违规添加情况时有发生。因此,亟需建立一种快速、准确的检测方法用于发酵食品中曲酸含量的测定,为食品安全监管部门对相关产品进行有效监测提供技术支持。

目前,针对曲酸的测定方法主要有光谱法^[7]^、电化学分析法^[8,9]^、高效液相色谱法^[10,11]^、气相色谱-质谱法^[12]^和液相色谱-串联质谱法^[13,14]^等。其中分光光度法难以准确定性;电化学方法重复性较差;气相色谱法、气相色谱-质谱法需要衍生化,操作较为繁琐;液相色谱法对基质复杂样品的定性存在局限性;高效液相色谱-串联质谱法无需衍生化,灵敏度好,定性能力强,适合痕量曲酸的测定。另一方面,发酵食品基质复杂,尤其是酱、酱油、腐乳等食品中含有大量的色素、蛋白质和油脂。目前大部分文献报道^[15,16]^主要集中于化妆品和单一食品基质如面粉等,罕见对复杂食品基质进行前处理的报道,且多采用直接提取测定,基质干扰严重,对仪器系统及色谱柱造成极大损害。有课题组^[17]^以乙酸锌和亚铁氰化钾为澄清剂用于沉淀蛋白质,但在共沉淀过程中可能会导致曲酸损失。固相萃取(SPE)技术具有有效将分析物和干扰物分离、有机溶剂消耗少、操作简便快速、易实现自动化等优点,是有机污染物净化富集的常见前处理方法。目前尚未见采用固相萃取方式净化食品来测定曲酸的方法。

本文通过固相萃取前处理,有效降低基质干扰,通过优化前处理条件和仪器条件,建立超高效液相色谱-串联质谱法(UPLC-MS/MS)测定发酵食品中曲酸含量,为相关监管部门对发酵食品中曲酸的监测提供了可靠的技术支撑。

## 1 实验部分

### 1.1 仪器、试剂与材料

Waters ACQUITY超高效液相色谱-Xevo-TQ-S质谱仪(美国Waters公司); N-EVAP氮吹仪(美国Organomation公司); XW-80A涡旋混匀器(中国其林贝尔仪器制造有限公司);高速冷冻离心机(中国英泰仪器有限公司);超声波清洗器(德国Elnma公司)。

甲醇、乙腈、正己烷(色谱纯,美国Merck公司);乙酸铵(色谱纯,美国ROE Scientific公司);甲酸(色谱纯,美国Fluka公司);乙酸锌、亚铁氰化钾、无水硫酸钠(优级纯,中国阿拉丁生化试剂有限公司);曲酸标准品(纯度≥98%,美国ROMER公司);内标U-^13^C_6_-曲酸(25 mg/L乙腈标准溶液,美国ROMER公司);实验用水为经Milli-Q净化系统处理的超纯水(美国Millipore公司)。PRiME HLB固相萃取柱(3 mL/60 mg,美国Waters公司)。酱油、酱、醋、酒、豆豉、腐乳等样品采自超市。

### 1.2 标准溶液的配制

称取10 mg曲酸标准品,用乙腈溶解、定容至10 mL,配制成质量浓度为1.0 g/L的标准储备液。移取0.10 mL标准储备液到10 mL容量瓶中,用乙腈稀释成质量浓度为10.0 mg/L的标准中间液。准确移取0.40 mL U-^13^C_6_-曲酸(25 mg/L乙腈标准溶液)于10 mL容量瓶中,用乙腈稀释成质量浓度为1.0 mg/L的内标中间液,这些标准溶液均在0~4 ℃下避光保存。

临用前,将初始流动相配制成质量浓度为0.5、1.0、2.0、5.0、10.0、25.0、50.0、100.0 μg/L的系列混合标准溶液,其中内标的质量浓度固定为10.0 μg/L。

### 1.3 样品前处理

将样品粉碎或搅拌均匀后,称取2 g试样置于50 mL离心管中,加入40 μL内标中间液(1.0 mg/L),加入2~4 g经马弗炉于550 ℃烘烤4 h后的无水硫酸钠,再加入10 mL 0.1%甲酸无水乙醇涡旋混匀,超声提取10 min, 10000 r/min离心5 min,取5.0 mL上清液于40 ℃氮吹至近干,加入2.0 mL超纯水复溶,待净化。

将上清液过PRiME HLB固相萃取柱,弃去前1 mL初滤液,收集续滤液,过0.22 μm水相滤膜,滤液用于UPLC-MS/MS测定。将未检出的样品视为空白基质,并在每类样品中选择一空白基质添加痕量曲酸标准溶液以确定检出限。

### 1.4 分析条件

#### 1.4.1 色谱条件

色谱柱:ACQUITY UPLC^®^ BEH C_18_ (100 mm×2.1 mm, 1.7 μm),流动相:A为甲酸-5 mmoL/L乙酸铵(1∶999, v/v)溶液, B为甲酸-乙腈(1∶999, v/v)溶液。梯度洗脱程序:0~1.0 min, 99%A; 1.0~1.5 min, 99%A~10%A; 1.5~3.0 min, 10%A; 3.0~3.1 min, 10%A~99%A; 3.1~5.0 min, 99%A。流速:0.3 mL/min,柱温:30 ℃,进样量:2 μL。

#### 1.4.2 质谱条件

离子化模式:电喷雾正离子(ESI^+^)模式;质谱扫描方式:多反应监测(MRM)模式;毛细管电压:2.0 kV;离子源温度:150 ℃;脱溶剂温度:500 ℃;脱溶剂气流速:1000 L/h;锥孔气流量:150 L/h。曲酸的质谱采集参数见表1。

**表1:** 曲酸和U-^13^C_6_-曲酸的质谱参数

Compound	Precursor ion (*m/z*)	Product ion (*m/z*)	CV/V	CE/eV
Kojic acid	143.1	97.3	15	23
		69.1^*^	15	25
U-^13^C_6_-kojic acid	149.1	73.2^*^	15	32

CV: cone voltage; CE: collision energy; * quantitative ion.

## 2 结果与讨论

### 2.1 色谱条件优化

发酵食品(如酱油、酱类)基质复杂,即便通过溶剂转换、固相萃取前处理,仍会存在部分极性杂质干扰分离,特别是曲酸作为强极性分子在反相色谱柱上的保留性能较差,因此选择具有较高保留性能和较好分离效果的色谱柱尤为重要。分别考察Inertsil ODS-3色谱柱(150 mm×4.6 mm, 5 μm)、ACQUITY UPLC BEH HILIC色谱柱(50 mm×2.1 mm, 1.7 μm)、ACQUITY UPLC BEH Amide色谱柱(150 mm×2.1 mm, 1.7 μm)、ACQUITY UPLC HSS T3色谱柱(150 mm×2.1 mm, 1.8 μm)和ACQUITY UPLC^®^ BEH C_18_色谱柱(100 mm×2.1 mm, 1.7 μm)对曲酸的分离效果。结果表明,Inertsil ODS-3色谱柱对曲酸的保留性能最好,出峰时间为4 min左右;Inertsil ODS-3色谱柱具有高纯度的球形硅胶填料、对称的粒径分布和较优的端基封尾处理技术,对极性化合物保留性能好,但其为普通液相色谱柱,在超高效液相色谱体系中容易导致峰形变宽。HILIC色谱柱使用未键合的亚乙基桥杂化(BEH)颗粒,仅对碱性极性化合物保留效果好,对酸性化合物保留较差,尤其在起始流动相为高比例水相的条件下,会出现峰形较宽的现象。Amide色谱柱为亲水作用色谱柱,其使用三键键合的酰胺基键合相以增强对极性物质的保留,但在试验中曲酸在l min内出峰,保留较弱。T3色谱柱适用于反相色谱分离中对极性化合物的保留,其低配位密度的C_18_烷基链使得分析物更容易进入颗粒材料的孔结构中,对曲酸保留较好,但峰形较差,且基线较高。BEH C_18_色谱柱采用BEH颗粒,适用pH范围更宽,具有出色的低pH稳定性,出峰时间适宜,峰尖锐且对称,因此最终选择ACQUITY UPLC^®^ BEH C_18_色谱柱用于曲酸的分离。

流动相的选择既要考虑色谱的分离效果,同时还需兼顾待测组分进入质谱的离子化效率,以获得最佳的分辨率和灵敏度。考察了乙腈与不同的水相(超纯水、5 mmol/L乙酸铵溶液、甲酸-5 mmol/L乙酸铵(1∶999, v/v)溶液)作为流动相对曲酸分离效果的影响。结果发现,超纯水作为流动相时分离效果较差,具有杂峰;加入5 mmol/L乙酸铵后,峰形得到明显改善,但仍有拖尾现象;加入甲酸后峰形对称,这是因为曲酸是二元弱酸,在酸性条件下易形成游离的分子,可有效改善拖尾现象,使色谱的分离效果变好,同时酸性条件下可以加强正离子的离子化效率。之后分别比较了加入0.1%、0.2%、0.3%、0.4%、0.5%甲酸对曲酸分离效果的影响。结果表明,在加入甲酸后,峰形均有所改善,较高体积分数的甲酸有利于降低基线,峰形更好。此外,在有机相和水相内均加入甲酸可使基线更加平稳。考虑到甲酸体积分数对色谱柱寿命的影响,最终选择甲酸-乙腈(1∶999, v/v)溶液和甲酸-5 mmol/L乙酸铵(1∶999, v/v)溶液作为流动相。

### 2.2 质谱条件优化

曲酸的吡喃环上连接有酚羟基和羟甲基,易于得到H^+^形成[M+H]^+^型分子离子峰,因此本实验选用ESI^+^模式。采用流动注射的方式,对曲酸进行母离子全扫描,确定曲酸母离子*m/z* 143.1和U-^13^C_6_-曲酸母离子*m/z* 149.1。随之进行子离子扫描,确定两个特征碎片离子,优化碰撞能量,并选择丰度较高的子离子为定量离子。在优化后的最佳色谱条件和质谱条件下,曲酸和U-^13^C_6_-曲酸的MRM色谱图见图1。

**图1:**
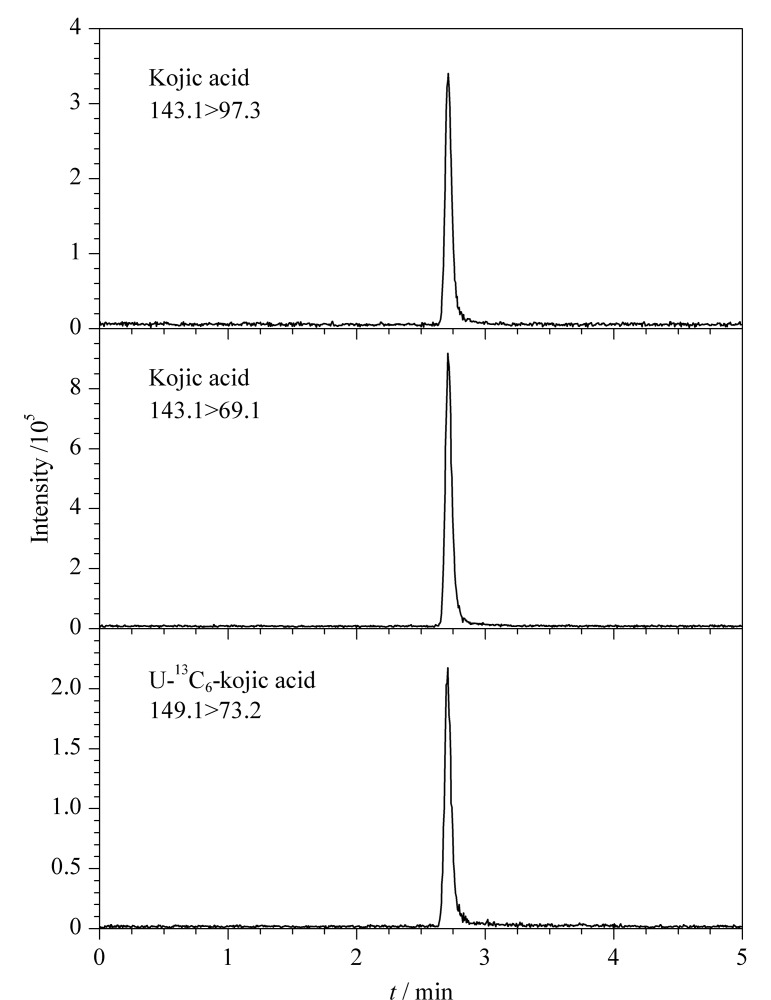
曲酸和U-^13^C_6_-曲酸的MRM色谱图

### 2.3 前处理条件的优化

#### 2.3.1 提取步骤的优化

曲酸为极性弱酸,易溶于水和亲水性较强的有机溶剂如乙醇、乙腈等^[18,19]^。此外,发酵食品中含有大量的盐分、油脂和蛋白质,为了便于后续净化浓缩,将样品脱水处理后用有机溶剂提取,一方面便于浓缩和溶剂转换,降低溶剂效应;另一方面可有效减少提取液的含盐量。分别考察了无水硫酸镁和无水硫酸钠的除水效果,结果表明,无水硫酸钠的除水效果更好。等质量无水硫酸镁的吸水能力虽然比无水硫酸钠强,但其吸水过程放热明显,不利于热敏性曲酸的提取,且无水硫酸镁在吸水过程中会形成坚硬的外壳附着在样品上,导致提取率显著降低。实验根据样品实际情况选择加入量,对于液态样品,无水硫酸钠的加入量应能使样品失去流动性为宜。

实验比较了不同体积分数(0、0.1%、1%、2%)的甲酸配合不同有机溶剂(甲醇、乙腈、无水乙醇)作为提取溶剂的提取效果,结果如图2所示。实验结果表明,在有机溶剂中添加甲酸可使曲酸的提取效果得到显著改善,但增大甲酸体积分数对回收率无明显影响;甲酸-无水乙醇系列溶液对曲酸的提取效果最佳,回收率为94.6%~98.5%,其次是甲酸-甲醇系列溶液(85.3%~87.4%)和甲酸-乙腈系列溶液(89.6%~92.3%)。因此,后续实验选用0.1%甲酸无水乙醇为提取液。此外,曲酸对光和热敏感,在这些环境下容易分解、氧化,因此前处理过程应尽量避光。

**图2:**
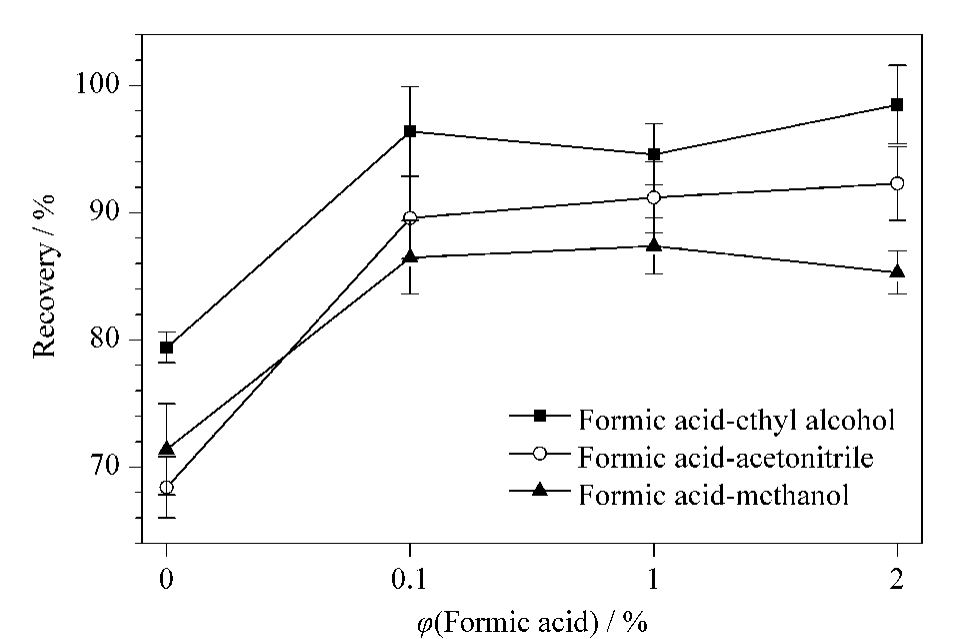
提取液种类及甲酸的体积分数对曲酸回收率的影响

#### 2.3.2 SPE条件的优化

采用SPE方式净化样品,通过吸附干扰杂质使待测物流出,实验分别考察了Oasis HLB(3 mL/60 mg)、PRiME HLB、C_18_(3 mL/500 mg)净化柱对待测物的净化效果。结果表明,PRiME HLB柱对曲酸的回收率最高,绝对回收率可达86.2%,其次是Oasis HLB柱,回收率为68.4%, C_18_柱对曲酸的回收率最低。PRiME HLB柱可以有效吸附样品中的非极性干扰物如蛋白质、脂肪、磷脂和色素等,降低基质效应,提高回收率。考虑到前处理方法的简便性和普适性,最终选用0.1%甲酸无水乙醇提取,经氮吹浓缩后用超纯水复溶,过PRiME HLB柱净化。

### 2.4 基质效应

通过比较基质匹配标准曲线与溶剂标准曲线来评价基质效应(ME), ME=(基质匹配标准曲线斜率/溶剂标准曲线斜率-1)×100%。当|ME|<20%时为弱基质效应,当20%≤|ME|≤50%时为中等基质效应,当|ME|>50%时为强基质效应;ME为负值时表示基质抑制效应,正值表示基质增强效应^[20]^。结果表明,醋和酒类基质效应弱,ME值分别为4.05%和5.57%;而酱类呈现强基质抑制效应,ME值为-59.7%;腐乳、豆豉、酱油为中等基质抑制效应,ME值分别为-46.8%、-44.2%、-33.9%。这些基质因含有大量的盐、色素、短肽及有机酸等大分子极性杂质,存在较强的基质抑制效应。因此,本方法采用同位素内标定量法降低基质带来的影响。

### 2.5 方法学考察

#### 2.5.1 线性范围、检出限和定量限

在确定的最佳测定条件下,曲酸在5.0~100.0 μg/L范围内呈现良好的线性关系,以曲酸与内标的峰面积比值为纵坐标(*y*),曲酸的质量浓度为横坐标(*x*, μg/L)绘制标准曲线并计算回归方程。回归方程为*y*=0.1303*x*-0.0048,相关系数(*r*)为0.9994。在空白基质中添加痕量曲酸标准溶液,以仪器信噪比(*S/N*)为3时所对应的标准溶液质量浓度为仪器检出限, *S/N*为10时所对应的质量浓度为仪器定量限。根据前处理步骤获得曲酸在腐乳、豆豉、酱、酱油、醋、酒中的方法检出限分别为5、5、5、3、2、2 μg/kg,方法定量限分别为15、15、15、9、6、6 μg/kg。

与文献相比,该方法的检出限优于其他采用类似检测技术的方法,见表2。此外,目前大部分的报道采用纯水或有机溶剂直接提取测定,简单快捷,但直接提取容易导致严重的基质干扰,高油性食品甚至会对仪器系统及色谱柱造成损害。本方法在转换溶剂后直接采用固相萃取柱净化,无需额外的淋洗和洗脱步骤,操作简便。

**表2:** 本方法与其他方法的比较

No.	Pretreatment	Technique	LOD/ (μg/kg)	Ref.
1	carbon past electrode	square-wave voltammetry	28.4	[1]
2	methanol ultrasonic extraction	HPLC	700	[10]
3	80% ethanol aqueous solution-acetonitrile (1∶1, v/v) vortex extraction	HPLC-MS/MS	25	[13]
4	diluted with water, and deproteinized by the deposition with zinc acetate	HPLC-MS/MS	30-800	[18]
	and potassium ferrocyanide			
5	water ultrasonic extraction	UPLC-MS/MS	30	[21]
6	PRiME HLB solid-phase extraction purification	UPLC-MS/MS	2-5	this method

#### 2.5.2 回收率和精密度

以腐乳、酱油、豆瓣酱、醋、酒为典型样品,在样品中分别添加低、中、高3个水平的曲酸标准溶液,按1.3节方法进行前处理并分析,考察方法的回收率、相对标准偏差(RSD)及日内、日间精密度,每个添加水平平行测定6次。结果如表3所示,曲酸的回收率为86.8%~111.7%,RSD为1.8%~4.3%,日内精密度为1.0%~7.9%(*n*=6),日间精密度为2.7%~10.2%(*n*=5)。

**表3:** 方法的回收率和日内、日间精密度

Sample	Background/ (μg/kg)	Spiked level/ (μg/kg)	Recovery/ % (*n*=6)	RSD/ % (*n*=6)	Intra-day RSD/ % (*n*=6)	Inter-day RSD/ % (*n*=5)	
Fermented bean curd	ND	15.0	89.4	2.7	4.1	8.9	
		30.0	94.7	2.3	3.8	5.7	
		60.0	95.7	1.9	2.9	4.3	
Soy sauce	29.9	15.0	109.5	3.1	1.2	2.9	
		30.0	111.7	2.1	1.0	7.8	
		60.0	108.8	2.8	7.9	3.8	
Sauce	160.1	80.0	89.5	2.9	1.2	7.1	
		160.0	93.1	3.6	3.9	5.9	
		320.0	88.7	2.5	4.4	2.7	
Vinegar	242.1	120.0	101.8	4.1	5.6	8.4	
		240.0	92.9	4.3	3.5	6.9	
		480.0	86.8	2.6	5.2	7.3	
Liquor	61.2	30.0	92.9	3.8	4.7	10.2	
		60.0	96.4	3.4	4.3	7.3	
		120.0	93.7	1.8	5.6	5.8	

ND: not detected.

### 2.6 实际样品检测

采用本方法对酱油、酱、醋、酒、豆豉、腐乳等240份食品样品中的曲酸含量进行了测定,发现醋的检出率最高,为63.3%,曲酸含量为41.7~2272 μg/kg,其次为酒、酱、酱油和豆豉,检出率分别为40.3%、26.8%、17.7%和11.5%,曲酸含量为5.69~160 μg/kg。腐乳样品中仅有1份检出曲酸。典型样品的MRM色谱图见图3。曲酸是曲霉属和青霉属真菌发酵的代谢产物,可以在发酵过程中天然产生,因此无法判断样品中的曲酸是否为人为添加,但发酵食品中曲酸的存在及其带来的风险值得相关监管部门关注与评估。

**图3:**
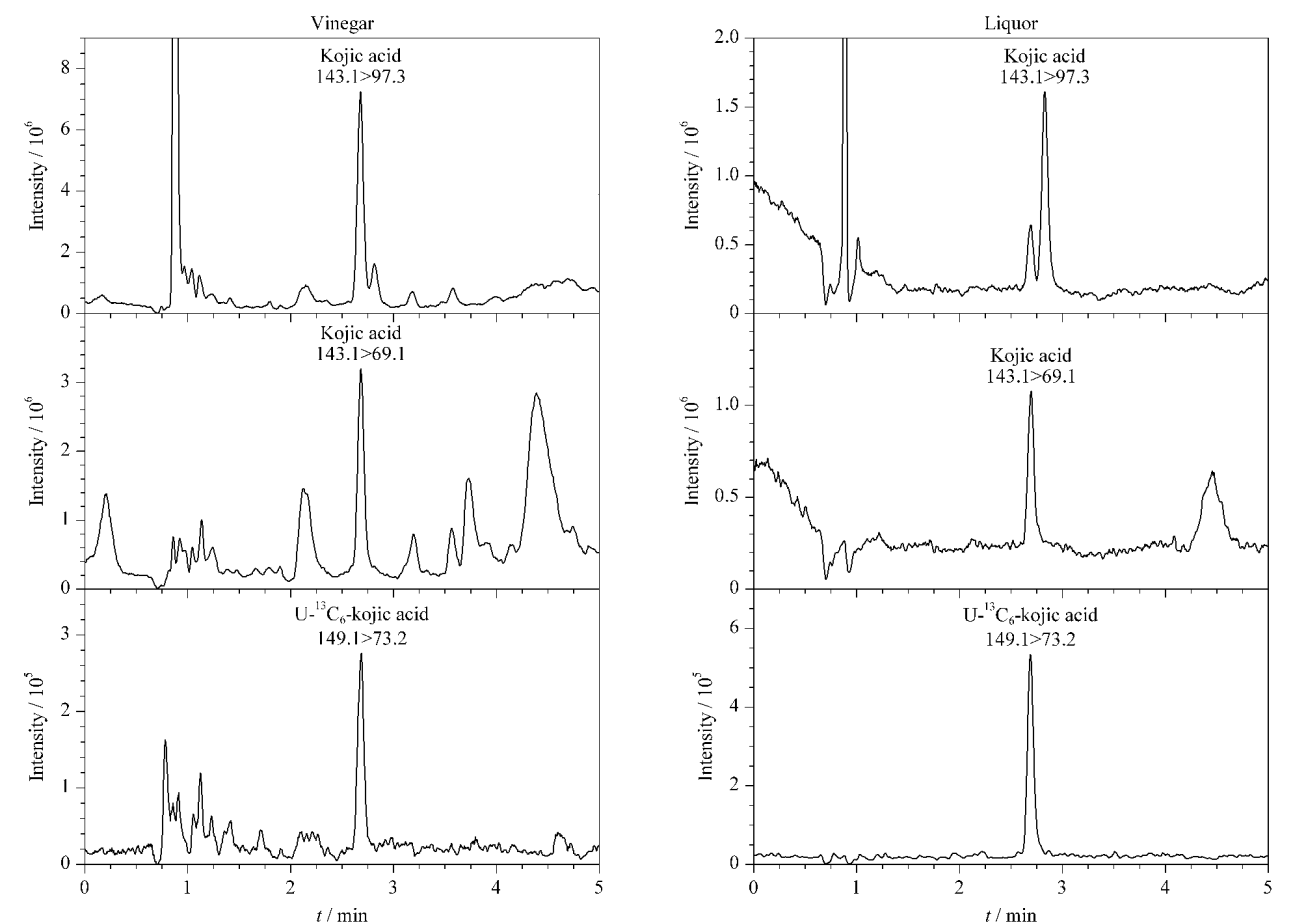
典型样品的MRM色谱图

## 3 结论

建立了发酵食品中曲酸的固相萃取-超高效液相色谱-串联质谱测定方法。该方法通过溶剂转换、固相萃取柱净化和内标法定量,有效克服了发酵食品中复杂基质带来的基质抑制效应,显著提高了方法灵敏度。固相萃取柱无需活化平衡,将提取液过柱、过膜后直接测定,简便快捷。该方法可用于发酵食品中曲酸的日常检测,为大规模监测曲酸含量提供了强有力的技术支持。
